# Sociodemographic characteristics of mother’s population and risk of preterm birth in Chile

**DOI:** 10.1186/1742-4755-10-26

**Published:** 2013-05-16

**Authors:** Paulina O López, Gérard Bréart

**Affiliations:** 1 INSERM, UMR S953, Recherche épidémiologique en santé périnatale et santé des femmes et des enfants, Hôpital Tenon, Paris F-75020, France; 2 Université Pierre et Marie Curie, UMR S 953, Paris, F-75005, France; 3 Universidad de Valparaíso, Escuela de Obstetricia y Puericultura, Valparaíso, Chile

**Keywords:** Preterm birth, Maternal population, Risk factor, Sociodemographic characteristics

## Abstract

**Background:**

Preterm birth is a global problem in Perinatal and infant Health. Currently is gaining a growing attention. Rates of preterm birth have increased in most countries, producing a dramatic impact on public health. Factors of diverse nature have been associated to these trends.

In Chile, preterm birth has increased since 90. Simultaneously, the advanced demographic transition has modified the characteristics of woman population related to maternity.

The principal objective of this study is to analyze some sociodemographic characteristics of the maternal population over time, and their possible association to rates of preterm birth. The second aim is to identify groups of mothers at high risk of having a preterm child.

**Methods:**

This population-based study examined all liveborn singletons in Chile from 1991 to 2008; divided in three periods. Preterm birth rates were measured as % births <37 weeks of gestation.

Logistic regression assessed the risk of preterm birth associated with mother’s age, parity, and marital status, expressed as crude and adjusted odds ratios.

**Results:**

Over time, rates of preterm birth increased in overall population, especially during the third period (2001–2008). In the same time, characteristics of maternal population changed: significant increase of extreme reproductive ages, significant decrease in parity and increase in mothers living without a partner.

Risk of preterm birth remained higher in groups of mothers: <18 and >38 years of age; without a partner; primiparas and grandmultiparas. However, global increase in preterm birth was not explained by the modification of socio demographics characteristics of maternal population.

**Conclusions:**

Some socio demographic characteristics remained associated with preterm birth over time. These associations allowed identifying five groups of mothers at higher risk to have a preterm child in the population.

Increase in overall preterm birth affected all women, even those considered at “low sociodemographic risk” and the contribution of more recent period (2001–2008) to this increase is greater.

Then, studied factors couldn’t explain the increase in preterm birth. Further research will have to consider other factors affecting maternal population that could explain the observed trend of preterm birth.

## Background

Preterm birth (PTB) is gaining increasing attention. During the four last years, several call for action and for research has been made throughout the systematic revue of worldwide incidence/prevalence and also throughout the series of Global Reports [1-4].

The seven Global Reports on preterm birth and stillbirth, issues of Conference on Preterm birth and Stillbirth on may 2009 (USA), estimated that 13 million babies are born before 37 completed weeks of gestation annually. Later, in the Global Action Report on Preterm Birth: Born Too Soon (WHO 2012), the number of preterm birth increased to 15 million annually.

Rates (% births <37 weeks of gestation), are generally higher in low- and middle income countries than high income countries. In these countries is born 92% of all preterm birth and 99% of them die. Rates are also increasing in most of these countries [5].

Multiple factors may be associated with increase in PTB, among them, socio demographics changes in maternal population such as increased maternal age and delayed primiparity [6,7]. Nevertheless, sociodemographic characteristics of women, as age, parity, marital status, age at first delivery and fertility rates, often have been the object of study by economists [8] and demographers [9,10]. Few studies has addressed their impact on obstetrical and perinatal outcomes, among them, researches in France (Unité 953 of INSERM) since 70’s, have reported that certain maternal conditions could affect maternal risk and could be associated with higher rates of preterm birth, with low birth weight and with fetal growth restriction [11,12]. Consequently, sociodemographic factors have been evaluated since 70’s through periodic surveys which monitor maternal and perinatal health [13,14].

Globally, few countries have been assessed the maternal sociodemographic factors over time as Finland and Sweden who have monitored the maternal population through studies in birth cohorts [15,16]. Those countries have also demonstrated that certain maternal characteristics are associated with higher risk of PTB and they showed that these characteristics may vary significantly over time.

Chile is a middle income country where the PTB rates have significantly increased [17]. This increase has been recorded in a context of demographic and epidemiological transition (which began in the 1960s) [18]. This process has led to modifications in some characteristics of the population of women (similar to those observed in some more developed European countries in the 1970s) including a diminution of fecundity rate from: 2.2 children per woman in 1996 to 1.9 children per woman in 2004; a decrease rate of marriage from 19.6 per 1000 in 1996 to 15.1 per 1000 in 2004. During this process it has also been observed an increase of employment and higher education and finally delayed maternal age at first children [9].

In Chile, maternal and perinatal health constitute a major focus of public health policies and PTB is a health priority [19]. However, the determinants of preterm birth in Chilean population are partially known. Therefore we cannot yet formulate primary and secondary prevention strategies based on specific characteristics of either women or mothers.

The objective of present study is firstly: to examine sociodemographics characteristics of maternal population in the past two decades, then, to analyze their association with the risk of PTB, and finally to identify groups at higher risk of giving birth to a premature child.

## Methods

### Study design and population study

This population-based observational study of singletons liveborn in Chile (1991–2008) examined 4 475 444 births recorded in the National Livebirth Database in Chile. The Chilean database is the official source of health statistics about live births in the country; it undergoes systematic verification from hospital sources. It does not impute any data. Data are available under demand of the researcher [20].

Data come from the birth certificate completed by the physician or midwife after delivery. This certificate is sent by the hospital to the Civil Registry. In Chile, overall, 99.8% of births occur in hospitals and clinics, with professional assistance (obstetricians, physicians or university-trained midwives) [19]. Live births at home are also recorded in the database. Stillbirths, however, are not.

The criterion for registration of live births in Chile is the same as that recommended by the World Health Organization (WHO): either a gestational age of 22 weeks or more, or a birth weight of 500 g or more [21].

According to WHO recommendations, birth weight is measured in grams [22] and gestational age (WGA) is calculated at the first prenatal visit and it is recorded in weeks of gestation from the last menstrual period. Later on gestational age is confirmed first by clinical examination and then by the first-trimester fetal ultrasound.

### Excluded data

From the initial complete set of singleton births, were excluded 636 observations (0.01%) before 22 WGA and 640 (0.01%) with a birth weight < 500 g. Subsequently, were excluded 815 (0.02%) ≥ 44 weeks WGA, and 5 850 (0.13%) with missing data. Moreover, were eliminated 75 076 observations (1.7%) with errors of classification of GA. These errors were detected by application of Tukey’s rule [23], by week of GA, to the distribution of length and birth weight. This method was selected after an exploratory analysis of the data base and also after examination of effect of these exclusions on the final distribution. Finally, these exclusions did not modify the observed trends [24].

### Outcome criteria

The principal outcome under study is preterm birth defined as births before 37 WGA. It was analyzed as overall rate (%).

### Maternal factors

Three maternal social and demographic characteristics were considered to be explanatory variables of PTB: namely age; parity and marital status.

Age was analyzed in years: <18 (adolescent or younger mothers), 18–38, and >38 (considered as older mothers).

Mothers younger than 18, are known as a group at high psychosocial obstetrical and fetal risk. Legally, in Chile they have not reached the majority of age.

Mothers older than 38 are considered by many authors as a group at high maternal and fetal risk [25]. In Chile, the increased risk in this group regarding premature births have been reported in recent studies [26].

Parity was classified as primiparity (first delivery), multiparity (one to four preceding deliveries), and grand multiparity (five or more preceding deliveries).

Finally, marital status was considered: one category including those living with a partner (legally married or not), and the other not living with a partner.

### Statistical analyses

At the beginning of this study, the period from 1990 to 2008 represented at that point, all the recorded information in the National data base. It was excluded only the first cohort (1990) when process of computerized recording was just started.

In order to observe the probable influence of health policies for mothers and child and also the changes in perinatal care, the period of observation was stratified in three periods. These periods are also in concordance with the progressive use of fetal ultrasound to estimate gestational age. Thus, during the first period 1991–1995 (P1), ultrasounds were performed for around 65% of pregnant women receiving prenatal care; during the second period 1996–2000 (P2), when the National Women’s Health Program was established, the use of first-trimester fetal ultrasound increased still further (75% of pregnant women receiving care). Finally, during the third period 2001–2008 (P3), ultrasound became routine, practiced for more than 90% of the pregnant women receiving prenatal care.

### Measurement of the risk associated to sociodemographic factors

– Prevalence of PTB and absolute risk

– Epidemiologically, % of PTB by period corresponds to rates of prevalence of PTB. The prevalence/100 = absolute risk. Absolute risk is the probability of disease in presence of the factor.

Odds Ratio and relative risk

Odds ratio (OR), is a relative measure that quantifies the strength of the disparity of exposure to factor between two groups (in this study: group of mothers with premature child is compared, by exposure to factor, to mothers without premature child).

When the prevalence of the disease is low (<10%), the value of OR is very close of the Relative Risk [27].

The association between each sociodemographic characteristic and PTB was evaluated by crude and adjusted OR and its Wald confidence interval from the logistic models of regression. Adjusted model allows take into a count the potential confounding effect of explanatory variables [28].

In logistic models of regression, the level of significance for *P* < 0.05, reference period was first period (initial prevalence) and the reference classes for the socio demographic characteristics were: 18–38 years, living with partner, and multipara.

A “period” variable in three classes (P1, P2, and P3) was generated, it was considered as an explanatory variable included in adjusted logistic models.

Statistical analyses were performed using STATA software SE 12.0

### Ethical considerations

This is an epidemiological and observational study. This is not an experimental design. The population database was provided under authorization of Statistical Section of Ministry of Health (DEIS MINSAL) [20] and data are not linked with medical or clinical files. Since the individual identification of women is not available, patient’s consent is not possible.

Primarily, the aim of this research is in compliance with the Helsinki Declaration with a reasonable likelihood of benefit to the population studied.

## Results

In all, 83 017 observations (1.85%) were removed from the initial set. Table 1 shows that the absence of these groups did not modify the observed trends, but it required a specific analysis. Some results from that specific group of births are: mothers with missing values of GA had more often the characteristics associated to PTB such as: not living with a partner: 54.9% versus 48.4% in all population, age >38 years old: 6.8% versus 4.3% in all population and grand multiparity: 9.1% versus 1.5% in all population, (data not showed). Also, births with missing values of BW had higher rates of PTB: (55.7%).

**Table 1:** Rates of preterm births by period before and after exclusions

**Periods**	**1991-1995**	**1996-2000**	**2001-2008**
**Rates**	**%**	**%**	**%**
Preterm births before excluded values	4.70	4.90	5.90
(n)	(1 354 711)	(1 258 750)	(1 861 983)
Preterm births after excludes values	4.42	4.71	5.65
(n)	(1 323 021)	(1 232 824)	(1 836 582)

Chile, Singletons Live Births, 1991-2008.

### Trend of preterm birth

From the final set for analyses, (N = 4 392 427), we can observe a highly significant increase (p <0.001) in the rates of PTB by period (Table 2).

**Table 2:** Rates of preterm births by period in Chile

**Periods**	**1991-1995**			**1996-2000**			**2001-2008**			
**N = 4 392 427**										
	**Rate %**	**95% CI**^**a**^	**(n)**	**Rate %**	**95% CI**	**(n)**	**Rate %**	**95% CI**	**(n)**	***P***^***b***^
Preterm Births^c^	4.42	[4.38,4.45]	1 323 021	4.71	[4.67,4.75]	1 232 824	5.65	[5.62,5.69]	1 836 582	< 0.001

Singletons Live Births, 1991–2008.

^a ^95% Confidence interval.

^b^Pearson’s chi-square test between periods.

^c^Preterm births in weeks of gestational age (WGA).

### Maternal socio demographics characteristics over time

Table 3 shows the following modifications in the maternal socio demographics characteristics: an increase of percentage of younger mothers. At the same time the number of older mothers rose continuously, particularly during the last period.

**Table 3:** Mothers’ characteristics throughout three periods

**N**	**1991-1995**		**1996-2000**		**2001-2008**		^**a**^**P1-P2**	**P2-P3**	**P1-P3**
**4 392 427**	**1323021**		**1232824**		**1836582**				
**Age**	**%**	**n**	**%**	**n**	**%**	**n**	***P***^***b***^	***P***	***P***
<18	5.6	74 659	7.2	88 197	7.1	130 035	*<0.001*	*<0.014*	*<0.001*
(292 891)									
18-38 (3 910 271)	91.0	1 204 514	88.8	1 095 027	87.7	1 610 730	*<0.001*	*<0.001*	*<0.001*
>38	3.3	43 848	4.0	49 600	5.2	95 817			
(189 265)							*<0.001*	*<0.001*	*<0.001*
**Marital Status**									
Living with partner	62.1	821 803	54.9	544 980	42.3	775 989			
(2 142 772)							*<0.001*	*<0.001*	*<0.001*
Not living with partner	37.9	501 218	45.1	446 808	57.7	1 060 593			
(2 008 619)							*<0.001*	*<0.001*	*<0.001*
**Parity**									
Primipara (1 849 118)	40.6	537 524	42.1	519 250	43.1	792 344			
							*<0.001*	*<0.001*	*<0.001*
Multipara (2 471 351)	57.2	756 413	56.2	692 944	55.7	1 021 994			
							*<0.001*	*<0.001*	*<0.001*
Grand Multipara	19.0	25 410	15.0	19 004	12.0	21 580			
(65 994)									
							*<0.001*	*<0.001*	*<0.001*

Chile, Singleton Live Births, 1991–2008.

^a^Periods : P1 : 1991–1995, P2 : 1996–2000, P3 :2001–2008.

^b^Pearson’s chi-square test with significance level for *P* < 0.05.

The group of mothers living without a partner increased steadily as well, especially during the last period when they became more numerous than those living with a partner.

Rates of primiparity remained steady between the first and second period but they increased later, while multiparas and grand multiparas decreased continuously. All these changes are statistically significant between periods.

### The prevalence of PTB and maternal socio demographics characteristics

Through the entire period the highest prevalence of PTB was seen among mothers younger than 18 years, those older than 38 years, primiparas, grand multiparas, and those not living with a partner (Table 4).

**Table 4:** Rates of preterm births by mothers’ socio demographic characteristics

**Periods**		**1991-1995**	**1996-2000**	**2001-2008**	^**a**^**P1-P2**	**P2-P3**	**P1-P3**	***Overall trend***
**Mothers’ characteristics**		**Rates (%) of Preterm births**^**b**^			***P***^***c***^	***P***	***P***	***P***
**Age**	<18	5.85	5.83	6.75	*0.84*	*<0.001*	*<0.001*	*0.002*
	18-38	4.22	4.49	5.41	*<0.001*	*<0.001*	*<0.001*	*<0.001*
	>38	7.19	7.65	8.35	*<0.001*	*<0.001*	*<0.001*	*0.001*
**Marital Status**	Living with partner	4.13	4.62	5.60	*<0.001*	*<0.001*	*<0.001*	*<0.001*
	Not living with partner	4.89	4.87	5.73	*0.66*	*<0.001*	*<0.001*	*0.007*
**Parity**	Primipara	4.62	4.95	5.93	*<0.001*	*<0.001*	*<0.001*	*<0.001*
	Multipara	4.17	4.47	5.41	*<0.001*	*<0.001*	*<0.001*	*<0.001*
	Grand multipara	5.76	5.76	6.92	0.99	*<0.001*	*<0.001*	*0.001*

Chile, Singletons Live Births, 1991–2008.

^a^Periods : P1 : 1991–1995, P2 : 1996–2000, P3 :2001–2008.

^b^Preterm births: < 37 weeks of gestational age.

^c^ Pearson’s chi-square test.

### Identifying groups of mothers at higher risk of preterm delivery

The difference between mothers with sociodemographic risk factor for preterm delivery and without sociodemographic risk factor may be observed in Table 5 that shows crude and adjusted ORs (AOR) by period. In other words, each factor is associated with the risk independently of the other. In effect, regarding confidence intervals, there is not a significant variation between crude and adjusted measures of OR. Moreover, the OR remained relatively steady between the periods.

**Table 5:** **Crude and adjusted odds ratios of preterm birth**^**a **^**associated with mothers’ socio demographic characteristics**

**Periods**		**1991-1995**				**1996-2000**				**2001-2008**			
**Mothers’ characteristics**^**b**^		**Crude OR**	**CI [95%]**	**Adjusted OR**^**c**^	**CI [95%]**^**c**^	**Crude OR**	**CI [95%]**	**Adjusted OR**	**CI [95%]**	**Crude OR**	**CI [95%]**	**Adjusted OR**	**CI [95%]**
**Age**	<18	1.42	[1.38,1.47]	**1.26**	[1.22,1.30]	1.33	[1.29,1.37]	**1.24**	[1.21,1.28]	1.28	[1.25,1.31]	**1.20**	[1.17,1.23]
	18-38		1.0 Reference^d^				1.0 Reference				1.0 Reference		
	>38	1.76	[1.69,1.82]	**1.78**	[1.72,1.85]	1.76	[1.70,1.82]	**1.82**	[1.75,1.88]	1.59	[1.56,1.63]	**1.65**	[1.61,1.69]
**Marital Status**	Living with a partner		1.0 Reference				1.0 Reference				1.0 Reference		
	Not living with a partner	1.20	[1.18,1.22]	**1.16**	[1.14,1.18]	1.06	[1.04,1.08]	**1.02**	[1.00,1.04]	1.03	[1.02,1.04]	**1.17**	[1.14,1.20]
**Parity**	Primiparous	1.09	[1.08,1.12]	**1.07**	[1.05,1.09]	1.10	[1.08,1.12]	**1.11**	[1.09,1.13]	1.10	[1.09,1.12]	**1.12**	[1.11,1.14]
	Multipara		1.0 Reference				1.0 Reference				1.0 Reference		
	Grand multipara	1.38	[1.31,1.46]	**1.33**	[1.25,1.42]	1.30	[1.22,1.38]	**1.28**	[1.19,1.38]	1.30	[1.23,1.37]	**1.26**	[1.18,1.35]

Chile, Singletons live births, 1991–2008.

^a^Preterm births : < 37 weeks of gestational age.

^b^Reference classes: 18–38 years, living with partner, multipara.

^c^All adjusted OR ( for the others factors in table) with *P* < 0.001 for Wald’s chi-square.

Table 6 shows that the last two periods contributed to higher rates of PTB. Globally, after adjustment, increase of PTB is 6% in the second period while during the third period, the increase of PTB is 26% (data no shown).

**Table 6:** **Crude and adjusted or for preterm births**^**a **^**by period**

**Period**	**1991-1995**	**1996-2000**		**2000-2008**	
**OR**^**b**^		**OR**	**CI 95%**	**OR**	**CI 95%**
Crude OR	Reference	1.07	[1.06, 1.08]	1.30	[1.28,1.31]
		OR	CI 95%	OR	CI 95%
Ajusted OR	Reference	1.06	[1.05, 1.07]	1.26	[1.25,1.28]

Chile, Singletons live births, 1991–2008.

^a^Preterm births : < 37 weeks of gestational age.

^b^All crude and adjusted OR ( adjusted for age, parity and marital status) with *P* < 0.001 for Wald’s chi-square.

The association between PTB and period is highly significant. This association remains unchanged, after adjustment by maternal sociodemographics characteristics.

Obtained results allowed identifying five groups of mothers at higher risk of giving birth to a preterm child. Their risk absolute is measured by the prevalence, and their relative risk is given by OR.

The five groups may be described according to the direction of their trends, as follows:

– Groups whose numbers are rising in the population:

• Mothers older than 38 years (compared with mothers aged 18–38 years). Despite a weak diminution of OR during the third period, this group remained high both risks: the absolute and relative risk of having a premature child.

• Mothers younger than 18 years (compared with those aged 18–38 years). They keep the same statistical relative risk over the time.

• The mothers living without partner (compared with those living with a partner). Adjusted OR of PTB declined during the second period but then rose again from 2001 onward.

– Group whose number was falling in the population:

• Grand multiparas, (compared to multiparas). Their relative risk remained unchanged over the time.

– Group whose number was relatively stable through the observed time:

• The primiparas (compared to multiparas). Adjusted OR for PTB did not vary after 1996, although it remained higher than during the first period.

## Discussion

Over an 18-year period, the rate of PTB has increased significantly in this population database. At the same time, the sociodemographic profile of mother’s population has changed substantially: parity has decreased and the percentage of younger, older and mothers not living with a partner rose.

Mothers following these trends had a considerable high risk of give birth to preterm child over time. Moreover, the number of women belonging to such groups at risk has also increased markedly (except for grand multiparas).

All and every of the studied factors are not enough to explain the increase of PTB because increase in the rates of PTB affected the entire population including those mothers without sociodemographic risk. Also, the more recent period contributed significantly to this increase independently of these factors.

Probably the last period reveals the effect of other possible factors, not available in this data base, such as life style changes, new patterns of sexual behavior or family structure. Some of this factors have already been observed by other studies in Chile [29].

Furthermore, another factors reported in the literature, but not available in data base, should be determined in this population, such as the status and conditions of working mothers, exposure to environmental and occupational contaminants, consumption habits, and most generally, the health issues of women.

Moreover, these trends could be related, as well, to the increase in obstetric interventions (cesarean deliveries and induction of labor) established by public and private policy in order to reduce maternal and fetal risk. In fact, the rates of both cesarean deliveries and induction of labor have been increasing in Chile for three decades and are among the highest in South America [30,31]. Unfortunately, the modality of delivery is not available in data base and this is an important limitation for understanding the nature of PTB.

The observed trends over the past two decades may also be related to an increase number of birth of extremely preterm infants. This could in part be associated to obstetrical and perinatal interventions aimed at diminishing fetal mortality among them, the progressive use of systematic fetal USN of first trimester.

The results suggest that in Chile, early, accessible prenatal monitoring ensures the collection of good quality data for estimating gestational age. It has reported a steady level of agreement between ultrasounds performed during the first trimester and gestational dates estimated by last menstrual period (61.9% in 1994 and 65.6% in 2001).

The quality of GA might be expressed in this study by the low proportion of error of classification of GA throughout the observation period. These errors decreased over time, this is probably the principal effect of systematic fetal USN of first trimester since the second period. In this context, the contribution of USN to increase of PTB seems to be modest. In addition, the trend to increase of PTB is sustained and it remains beyond the systematization of USN.

In all, only 1.85% of births were excluded of the initial set. This proportion is lower than other studies in population data base [32,33]. But the excluded births did not have a random distribution, then the exclusion of these groups could have underestimated the association between maternal characteristic and PTB especially during the first period, nonetheless the effect on the rates seems to be insignificant since these exclusions did not affect the trends of PTB.

The Tukey’s rule, was also used by Arbuckle in Canada [34], this method has been recommended for data base with few errors [23].

Comparing with previous studies, the trends observed in the groups at highest risk are similar to those observed in studies of parity, marital status and older mothers in France [35] and England [11].

Similar results have been observed In Belgium [36], whit a marked increase in the frequency of known factors contributors to PTB such as: maternal age > 35, assisted reproduction, obstetric intervention. Also increased the number of women with one or more of these contributors, but rate of PTB had a significantly increase in both groups: women with and women without these factors. Thereby, the steady increase in preterm birth could not be explained by these factors.

In the same direction of these results, Haas in USA [37] has reported a modest contribution of sociodemographic factors to increase of PTB. He concludes that other factors could be more important such as health status and access to medical care both before and during pregnancy.

In Chile access to medical care during pregnancy is universal. Beyond this, the high — and still rising — proportion of adolescents mothers is the continuation of an unsolved public health problem in Chile [38].

Countries such as France [14] and Finland [39], which have implemented long-term policies aimed at preventing preterm birth via integral prenatal care and social policies to support motherhood, have succeeded in significantly reducing the risk of PTB in younger mothers. Chile has not done so. This result confirms the importance of preconception care, from a conceptual framework such as has been given in Born Too Soon [5].

Among the groups at highest risk, the observed increase in maternal age is a new public health challenge in terms of maternal and perinatal results, as it requires a greater focus regarding the need for medical interventions during pregnancy and delivery, for assisted reproductive technologies, and for other highly specialized services [40].

Chile reached in 2003 its lowest rate of maternal mortality 12.2/100.000 live births after years of successfully reducing its prevalence, nevertheless the prevalence has risen over the past five years to 19.8/100.000 live births in 2005. The authors claim that maternal age over 35 years is the principal explanatory factor of this result, due to concomitant chronic diseases affecting such group of mothers [41].

Unfortunately, one of the principal limitations of the study is our inability to assess other important factors like clinical maternal and fetal condition. Moreover the absence of information available about the mode of delivery prevents us from being able to distinguish between spontaneous and medically indicated PTB [9].

Concerning rates of multiple gestations in Chile, they are increasing; this trend is showed in a recent study made over the same period (1991–2008) [17]. This increase could also be associated to the maternal age and also to the increase of fertility treatments.

Concerning assisted reproductive technologies (ARTs), they are rising and it is related to the increase of maternal age and late primiparity. However, this group of births has not significantly contributed to the rate of prematurity. Indeed, the report including twenty years of ARTs in Chile shows that between 1991 and 2008 only 3910 children were born through ARTs. The contribution of this group to preterm birth in those twenty years is poor: 353 single preterm birth, 585 doubles preterm and 210 preterm births in triples [42].

The principal reason is that these techniques are not yet a large coverage because they are very high cost.

Another challenge is the group of mothers living without partner. This is the only group for which the risk is probably due to sociodemographic context; nonetheless this group is still largely unknown. Countries that have succeeded in reducing this risk have implemented measures to support these mothers and to reduce their psychosocial and economic vulnerability [43,44]; Chile, so far lacks policies in these respect.

Further studies may be suitable using the others available variables in data base, such as: socio demographics characteristics of father, maternal education level. This group of variables could guide the hypotheses about the socio economic condition as important contributor au PTB.

In this direction, the study of K Joseph gave an important orientation about the distribution of sociodemographics factor by socioeconomics levels.

At the same way, the evaluation of the risk associated with interaction between these factors appears useful in view of the multifactorial etiology of PTB and LBW. For the moment; little research exists in this domain, but Schempf, in the United States, observed an important increase in risk of PTB in both groups: primipara teenager and primipara older in Hispanic mothers [7].

### Implications

The main strength of this research is having allowed identify some sociodemographic factors of risk of preterm birth over time, as well as, allowed identifying the trends of mothers with such characteristics.

The results indicate the need to redirect resources toward specific primary and secondary prevention but also toward the research; otherwise the trends reported here might unfortunately continue over time. Measures for prenatal care appropriate to this “new profile of mother’s population” should be considered and implemented through education, organization of care and professional resources. Resources also should be intended to the whole population of the women.

The implication of this findings, both, from a clinical and public health perspective is particularly interesting to other middle-income country similar to Chile, or countries which are beginning the process of demographic transition. The results might aid in the analysis of challenges to be expected in the domain of preterm birth.

Unfortunately, this study does not provide a clear explanation for the sustained increase of these births over nearly two decades in Chile. This limitation is principally due to lack of available data. Then this study revealed the enormous need for information about obstetric care, the lifestyle of women, their work context and their reproductive choice between other factors not considered here.

These factors may affect all women and they could help explain the growing trend of the premature birth as epidemiological phenomenon currently affecting most countries in the world. The results support the necessity of use of research and action, formulated by the last global report Born Too Soon.

The first, in order to understand thoroughly the causes of increase of PTB, there is a need to strengthen existing data by collecting detailed information about events and clinical history during pregnancy and delivery.

Further progress in explaining the determinants beyond that were analyzed here will require an interdisciplinary approach that will take into account their diversity, their complexity and their probable interactions. Such approach could shed light on the role of qualitative factors, such as women’s lifestyles or family structure.

## Conclusions

Preterm birth has increased over the past two decades in Chile. Some sociodemographic characteristics of mothers are steadily associated with the risk of preterm birth over time. Such association has allowed identifying five groups of mothers at higher risk of PTB. The epidemiologic importance of these groups is changing over time through an increase in the number of women belonging to groups at higher risk of preterm delivery.

Despite of these specifics results, sociodemographics factors, by themselves, are not enough to explain the marked trends to increase in rates of PTB affecting the overall population.

Further research could be define the role of others underlying factors over time, that are probably widely affecting the population of mothers, especially during the most recent period.

## Competing interests

The authors declare that they have no conflicts of interest in regard to this research.

## Authors’ contributions

PL Designed the study, made possible the acquisition of data, analyzed and interpreted the data. GB Made substantial contributions to conception and design of this study, has been involved in drafting the manuscript and revised it, providing critical and important intellectual content. It has revised and approved the final version submitted for publication. Both authors read and approved the final manuscript.
